# Prescribing of adjuvant analgesics among patients in primary care and specialized pain clinic

**DOI:** 10.1192/j.eurpsy.2021.1167

**Published:** 2021-08-13

**Authors:** E. Malenkova, O. Zagorulko

**Affiliations:** Pain Clinic, Russian Scientific Centre of Surgery named after B.V.Petrovsky, Moscow, Russian Federation

**Keywords:** Antidepressants, Pain, chronic low back pain, Chronic Pain

## Abstract

**Introduction:**

Chronic low back pain (CLBP) is one of the most resistant pain conditions and is often combined with psychoemotional disorders [1].

**Objectives:**

To analyze the frequency of prescribing adjuvant analgesics among patients with CLBP by specialists of the outpatient department and specialized pain clinic.

**Methods:**

The prospective study included 269 patients (group 1) with CLBP treated in an outpatient department and 253 patients (group 2) of specialized pain clinic. We analyzed gender, age, duration, and severity of pain (using the visual analogue scale-VAS), frequency of prescribing anticonvulsants and antidepressants, as well as their combination in both groups. The data were analyzed with IBM SPSS Statistics.

**Results:**

Among the patients of both groups, women predominated (65.3% in group 1 and 57.2% in group 2). The average age was 61.8±14.5 and 58.9±12.7, in the first and second groups, respectively. The disease duration was longer in group 2 (6.8±3.9 years, and 4.5 ± 2.7 in group 1, p<0.05). Pain intensity was comparable in both groups (4.3±2.8 and 5.1±2.5, p<0.067 on VAS). Antidepressants there were prescribed 16.1% and 52.9%, p<0.05, anticonvulsants - 18.8% and 33.2 %, p<0.05, their combination - 2.2% and 13.8%, p<0.05 in the first and second groups, respectively.

**Conclusions:**

Adjuvant analgesics are more often prescribed to patients of specialized pain clinics. It may be associated with more severe descriptions of chronic pain syndrome, as well as insufficient awareness of modern approaches to the management in this category of patients by specialists in primary health care. References: 1.Zagorulko, Medvedeva Russ Pain J. 2019

